# HPLC-HESI-MS/MS Analysis of Phenolic Compounds from *Cynoglossum tubiflorus* Leaf Extracts: An Assessment of Their Cytotoxic, Antioxidant, and Antibacterial Properties

**DOI:** 10.3390/plants13060909

**Published:** 2024-03-21

**Authors:** Dhouha Dallali, Jawhar Fakhfakh, Cédric Paris, Nissaf Aoiadni, Stéphanie Philippot, Arnaud Risler, Mihayl Varbanov, Noureddine Allouche

**Affiliations:** 1 Laboratory of Organic Chemistry LR17ES08, Natural Substances Team, Faculty of Sciences of Sfax, University of Sfax, Sfax P.O. Box 1171, Tunisia; dallalidhouha89@gmail.com (D.D.); jawharfakhfakh@yahoo.fr (J.F.); 2 Université de Lorraine, LIBio, F-54000 Nancy, France; cedric.paris@univ-lorraine.fr; 3 Laboratory of Animal Eco-Physiology, Faculty of Sciences of Sfax, Sfax P.O. Box 1171, Tunisia; nissafaoiadni93@gmail.com; 4 Université de Lorraine, CNRS, L2CM, F-54000 Nancy, France; stephanie.philippot@univ-lorraine.fr (S.P.); arnaud.risler@univ-lorraine.fr (A.R.); mihayl.varbanov@univ-lorraine.fr (M.V.); 5 Laboratoire de Virologie, CHRU de Nancy Brabois, F-54500 Vandœuvre-lès-Nancy, France

**Keywords:** *Cynoglossum tubiflorus*, HPLC-HESI-MS/MS, antioxidant, antimicrobial, cytotoxicity

## Abstract

The current study aimed to investigate the chemical composition, antioxidant, antibacterial, and cytotoxic properties of three extracts (hexane, dichloromethane, and methanol) from *Cynoglossum tubiflorus.* The composition of the methanolic extract was elucidated using HPLC-HESI-MS/MS analysis. The antioxidant effect was examined using NO, DPPH, FRAP, and TAC assays. Antimicrobial activity was evaluated by broth microdilution using various bacterial strains such as *S. aureus*, *S. epidermidis*, *P. aeruginosa*, *E. coli,* and *K. pneumoniae*. Structural disruptions in Gram-positive bacteria were visualized using scanning electron microscopy (SEM). Cytotoxic effects were evaluated on human MRC-5 in culture according to the MTT assay. The outcomes suggest that methanol extract contained a high amount of phenolic compounds (254.35 *±* 0.360 mg GAE/g DE and 211.59 *±* 0.939 mg QE/g DE). By applying the HPLC-HESI-MS/MS analysis, 32 compounds were identified, including phenolic acids, flavonoids, lignans, and fatty acids. This extract showed strong antioxidant (IC_50_ = 0.043 ± 0.001 mg/mL) and antimicrobial (MIC = 156 µg/mL) activities. The SEM suggests that cells exhibited membrane distortions characterized by surface depressions and alterations in bacterial shape, including dents, when compared to untreated cells. The in vitro cytotoxicity effect on human MRC-5 cells showed no toxicity effects at a concentration of 600 µg/mL. In silico analysis predicted low toxicity for all tested compounds across four different administration routes. This research indicates that this plant could be explored as a powerful source of natural drugs to target pathogens, with applications in the food, pharmaceutical, and medical industries.

## 1. Introduction

Secondary metabolites present a wide diversity of therapeutic applications and biological properties and play an important role in human health, being beneficial in the prevention of various health problems [1]. Medicinal plants have long been used as therapeutic agents due to their richness in bioactive compounds. Indeed, opting for naturally derived drugs over synthetic ones can reduce the various side effects associated with the latter. According to the World Health Organization (WHO), approximately 80% of the global population uses alternative medicine for their healthcare needs, primarily relying on the active components of plant extracts [2]. Boraginaceae is a family of plants that includes a variety of shrubs, herbs, and trees, totaling about 130 plant genera and 2300 species [3]. The genus *Cynoglossum* L. belongs to the Boraginaceae family, which contains approximately 75 species found in temperate regions of Africa, Asia, and Europe [4]. The plants from the *Cynoglossum* L. genus are very uniform in their external morphology, placing these plants among the most difficult to study taxonomically [5]. Several plants within the *Cynoglossum* L. genus, such as *Cynoglossum lanceolatum Forsk*, are used in traditional Chinese folk medicine to address a range of conditions, including nephritis, periodontitis, acute submandibular lymphadenitis, and snake bites [6]. Additionally, species like *Cynoglossum cheirifolium*, *Cynoglossum creticum*, and *Cynoglossum zeylanicum* have garnered scientific recognition for their antioxidant, antifungal, enzyme-inhibiting, anti-inflammatory, analgesic, and hepatoprotective properties [4,7,8]. These plants contain abundant secondary metabolites, including alkaloids, polyphenols, and fatty acids, as documented by Ilhem et al. and Ranabhat et al. [9,10]. Among these plants, *Cynoglossum tubiflorus* has been studied in the quest for bioactive molecules. In a previous investigation, eight fructooligosaccharides were isolated from the root’s extracts of this plant, and they proved to have good in vivo antidiabetic activity [11]. In-depth exploration of this plant species drove the objectives of this investigation, which were to evaluate the antibacterial and cytotoxic properties of leaf extracts (hexane, dichloromethane (DCM), and MeOH) and to examine, for the first time, their phenolic compounds using HPLC-HESI-MS/MS analysis. The cytotoxic effect of extracts was evaluated in MRC-5 human cells according to the MTT test. Moreover, the antioxidant properties were evaluated using the free radical scavenging potential of 2,2-diphenyl-1-picrylhydrazyl (DPPH), nitric oxide activity (NO), ferric reducing antioxidant power (FRAP), and total antioxidant capacity (TAC) assays. The antimicrobial activity was assessed using the microdilution broth method. The scanning electron microscopy (SEM) was performed using a Hitachi S-4800 microscope, and secondary electron images were taken at low electron energies ranging from 1 keV to 2.5 keV. Finally, the chemical composition of the methanol extract was investigated by HPLC-HESI-MS/MS analysis.

## 2. Results and Discussion

### 2.1. Extraction

Table 1 shows the extraction yields of *Cynoglossum tubiflorus* leaves using different solvents. The extraction yield of methanol extract (1.68%) was higher than that of DCM (1.04%) and hexane (1.62%). This difference in yields could be attributed to the different affinities between the polarity of solvents and the chemical compounds of extracts [12].

### 2.2. Total Phenolic and Flavonoid Contents of Cynoglossum tubiflorus Leaves

Phenolic compounds, primarily flavonoids, are naturally occurring bioactive substances predominantly present in plants. They exhibit intriguing properties, including antioxidant and antimicrobial effects [13]. The findings presented in Table 2 suggest that the MeOH extract had the highest TPC (254.34 ± 0.360 mg GAE/g DE) and TFC (211.59 ± 0.939 mg QE/g DE) contents, followed by the DCM (138.16 ± 0.114 mg GAE/g DE and 118.90 ± 0.352 mg QE/g DE) and hexane (88.94 ± 0.171 mg GAE/g DE and 54.57 ± 0.586 mg QE/g DE) extracts. To the best of our knowledge, the *Cynoglossum tubiflorus* species has not been evaluated for its phenolic and flavonoid contents. However, various studies have reported that Boraginaceae species are abundant in flavonoids and phenolic compounds [3,14]. Furthermore, *Cynoglossum tubiflorus* was found to have higher levels of phenolic and flavonoid contents compared to *Cynoglossum cheirifolium* L. [9] and *Cynoglossum zeylanicum* [10]. It exhibited double the amounts of total phenolic content (TPC) and total flavonoid content (TFC). These findings underscore the importance of selecting this plant for further in-depth investigation.

### 2.3. Identification of Secondary Metabolites in the Methanolic Extract

**Figure 1:**
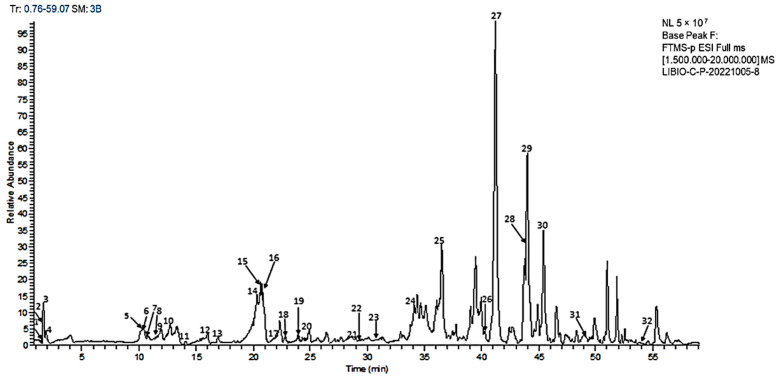
HPLC-HESI-MS/MS chromatogram, base peak (*m*/*z* 150–2000) of the leaf methanolic extract of *Cynoglossum tubiflorus* obtained with a reverse phase C18 column in negative mode ionization.

HPLC-HESI-MS/MS was used to identify the major secondary metabolites detected in the methanolic extract from *Cynoglossum tubiflorus*. Figure 1 illustrates the total ion mass chromatogram profile, while Table 3 presents MS/MS data for the tentatively identified substances.

#### 2.3.1. Characterization of Phenolic Acids

Compounds **3**, **4**, **8**, **10,** and **14** presented the same typical ion at *m*/*z* 191 (C_7_H_11_O_6_) corresponding to [quinic acid–H]^−^ [40]. Compound **3** was identified as quinic acid due to molecular ion [M-H]^−^ at 191 and other characteristic fragments *m*/*z* 173 [M-H-18]^−^, *m*/*z* 127 [quinic acid-64]^−^, *m*/*z* 109 [quinic acid-82]^−^, *m*/*z* 93 [phenol unit]^−^ [17]. Compound **4** corresponded to a quinic acid derivative with a pseudo-molecular ion [M-H]^−^ at *m*/*z* 533 and an MS^2^ fragment at *m*/*z* 191 (deprotonated quinic acid) [18]. Compound **8** was proved to be 1,5-dicaffeoylquinic acid with a molecular ion [M-H]^−^ at *m*/*z* 515 and MS^2^ fragment ions at *m*/*z* 353 [M-H-glucose]^−^, and *m*/*z* 191 (deprotonated quinic acid) [22]. 

#### 2.3.2. Characterization of Flavonoids

Compound **13** presented a molecular ion [M-H]^−^ at 593. This deprotonated molecular ion produced fragments at *m*/*z* 503 [M-H-90]^−^, 473 [M-H-120]^−^, and 383 [M-H-120-90]^−^. This compound was identified as 6,8-*C-*dihexosylapigenin [22,41]. Compound **15** showed a molecular ion [M-H]^−^ at 447 and typical fragment ions of C-glycosides *m*/*z* 357 [M-H-90]^−^ and 327 [M-H-120]^−^. This compound was identified as luteolin 8-*C-*hexoside [18]. Compound **19** exhibited a molecular ion [M-H]^−^ at 463 and its Ms^2^ fragmentation gave a base peak at *m*/*z* 301 due to the loss of glucose [M-H-162]^−^. Thus, compound **19** was deduced as quercetin-3*-O*-glucoside [29]. Compound **20** gave a [M-H]^−^ ion at *m*/*z* 577. Its MS^2^ spectrum gave a base peak at *m*/*z* 283 [M-H-162-132]^−^, attributed to the elimination of hexose and pentose moieties, respectively. Then, compound **20** was identified as lanceolarin [30]. Compound **11** presented the deprotonated molecule [M–H]^−^ at *m*/*z* 431 and underwent the loss of [M-H-46]^−^ (formate) to produce the ion at *m*/*z* 385; consequently, compound **11** was identified as a roseoside [18].

#### 2.3.3. Characterization of Lignans and Flavonolignans

Compounds **16** and **17** were identified as hydroxypinoresinol-4′-*O*-glucoside and cinchonain Ib, respectively. Hydroxypinoresinol-4′-*O-*glucoside produced a pseudo molecular ion [M - H]^−^ at *m*/*z* 535 and a base peak at *m*/*z* 373 corresponding to the loss of a hexose moiety [M - H-162]^−^ [26]. Cinchonain Ib is a flavonolignan found in different plant species. This compound generated an ion peak [M-H]^−^ at *m*/*z* 451 and its MS^2^ spectrum gave a base peak at *m*/*z* 341 [M-H-C_6_H_6_O_2_]^−^. These data were in accordance with a previous study [27].

#### 2.3.4. Characterization of Fatty Acids

Compounds **28**, **30**, **31,** and **32** were easy to interpret with exact masses of 293.2120, 295.2272, 277.2167, and 309.2793 as hydroxy-octadecatrienoic acid, hydroxy-octadecatrienoic acid, 15,16-dihydrotanshinone I, and eicosanoid acid, respectively [36]. Compound **24,** with an [M–H]^−^ ion at *m*/*z* 327 and MS^2^ fragments at 309, 291, 239, and 229, was identified as oxo-dihydroxy-octadecenoic acid (oxo-DHODE) [34,35]. Compound **25** was identified as trihydroxy-octadecenoic acid, considering its [M–H]^−^ ions at *m*/*z* 329 and its fragmentation pattern, previously described in the literature [36].

### 2.4. Antioxidant Activity

The antioxidant activity of the three *Cynoglossum tubiflorus* leaf extracts was determined using different complementary tests (DPPH, NO, FRAP, and TAC) due to their precision and simplicity.

#### 2.4.1. DPPH Assay

DPPH radical is stable at ambient conditions; it is largely used to evaluate the free radical scavenging activity of various natural substances. The IC_50_ values used to determine the antioxidant activity represent the quantity of sample necessary to trap 50% of a given concentration of free radicals. As shown in Table 4, the obtained results indicate that methanolic extract is a powerful inhibitor of DPPH given the fact that it had the lowest IC_50_ value (0.043 ± 0.001 mg/mL), followed by DCM (IC_50_ = 0.060 ± 0.001 mg/mL) and hexane extracts (IC_50_ = 0.383 ± 0.002 mg/mL). 

#### 2.4.2. Nitric Oxide Assay

The NO test was used to confirm the results obtained with DPPH. The obtained results (Table 4) disclosed an important NO chelation activity. The methanolic extract presented the highest NO chelation activity (IC_50_ = 0.046 ± 0.001 mg/mL), followed by DCM (IC_50_ = 0.121 ± 0.001 mg/mL) and hexane extracts (IC_50_ = 0.268 ± 0.09 mg/mL). 

#### 2.4.3. Ferric-Reducing Antioxidant Power (FRAP)

**Figure 2:**
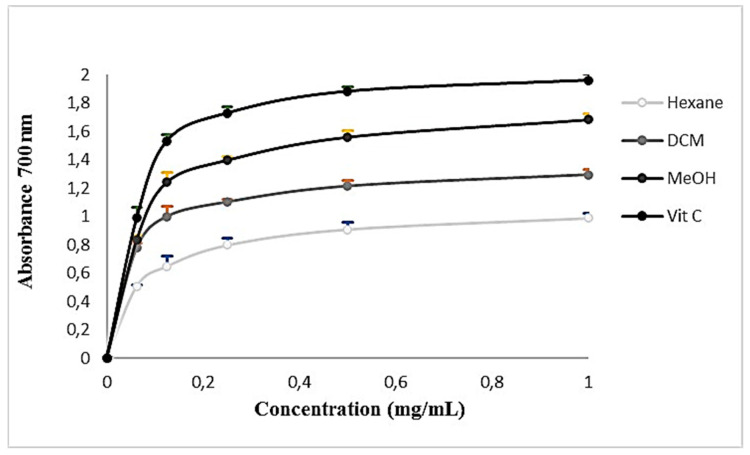
Reducing the power activity of *Cynoglossum tubiflorus* leaf extracts. Values are presented as mean ± SD (*n* = 3).

The ferric-reducing antioxidant power method relies on antioxidants’ capability to convert Fe^3+^ to Fe^2+^, forming a blue complex at low pH. As shown in Figure 2, the reducing power of extracts was proportional to the concentration. Increasing the concentration led to a corresponding increase in reducing power. According to the absorbance values obtained and compared to the positive control vitamin C, methanolic extract exhibited a higher reducing power compared to DCM and hexane extracts.

#### 2.4.4. Total Antioxidant Activity (TAC)

**Figure 3:**
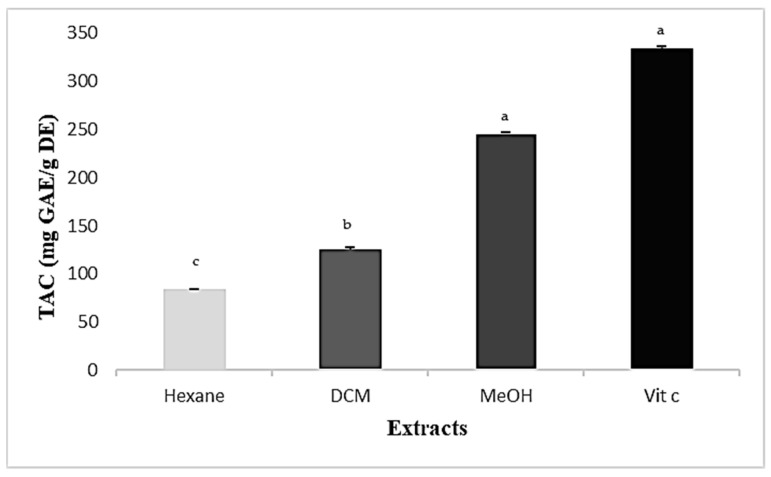
Total antioxidant capacity (TAC) of *Cynoglossum tubiflorus* leaves. Values are presented as mean ± SD (*n* = 3). The differences were analyzed using an ANOVA test for multiple comparisons using *p* < 0.05. ^a^: strong significance; ^b^: high model significance; ^c^: low significance.

The results arising from the total antioxidant capacity (TAC) test are presented in Figure 3. The methanolic extract had the highest antioxidant capacity (245.93 ± 1.801 mg GAE/g DE) compared to the DCM (125.81 ± 1.463 mg GAE/g DE) and hexane (83.97 ± 0.292 mg GAE/g DE) extracts.

The observed antioxidant activity of the methanolic extract from *Cynoglossum tubiflorus* leaves can be attributed to its abundant content of phenolic compounds and flavonoids, as suggested by previous studies [42]. In a previous research investigation, Ilhem et al. worked on a species from the genus *Cynoglossum* and reported good antioxidant activity for all the tested fractions, with IC50s ranging from 0.078 to 0.483 mg/mL [43]. However, in comparison with these results, *Cynoglossum tubiflorus* was found to be even more active in terms of antioxidant activity due to its richness in phenolic compounds, especially flavonoids. Phenolic compounds and flavonoids are well-known for their antioxidant properties, as they can neutralize free radicals and prevent oxidative damage in biological systems. The tested extracts demonstrated notable antioxidant potency through various mechanisms. Specifically, the methanol extract exhibited effectiveness in reducing Fe^3+^ to Fe^2+^, converting Mo^6+^ to Mo^5+^, and scavenging free radicals, which are known to pose significant health risks to humans [44].

### 2.5. Antibacterial Assay 

The obtained results reported in Table 5 demonstrated the susceptibility of the bacteria to the different extracts, with MIC values between 78–156 µg/mL for the Gram-positive bacteria and 312–625 µg/mL for the Gram-negative ones. Gram-positive bacteria are more susceptible to the extracts than Gram-negative ones, due to the structures of microorganisms. In fact, the Gram-negative bacteria possess an outer membrane that serves as a barrier to the passage of numerous molecules [45]. The hexane extract showed the most significant antibacterial activity against all tested strains, with the MeOH extract following closely behind. This observation may be attributed to the presence of natural compounds capable of intercalating within bacterial strains, thereby impeding their function. This activity may be due to chlorogenic acid and some of the various polyphenolic compounds, such as kaempferol and cirsimaritin, present in this extract (Table 1). In fact, several studies have shown the interesting effect of chlorogenic acid on bacterial strains such as *S. aureus*, *E. coli*, and *S. tiphymurium*. Further investigation into the action mode of chlorogenic acid against pathogens suggested that it significantly enhances the permeability of both the outer and plasma membranes, leading to disruption of their barrier functions and even causing slight leakage of nucleotides [46,47].

### 2.6. Electron Microscopy Assay 

**Figure 4:**
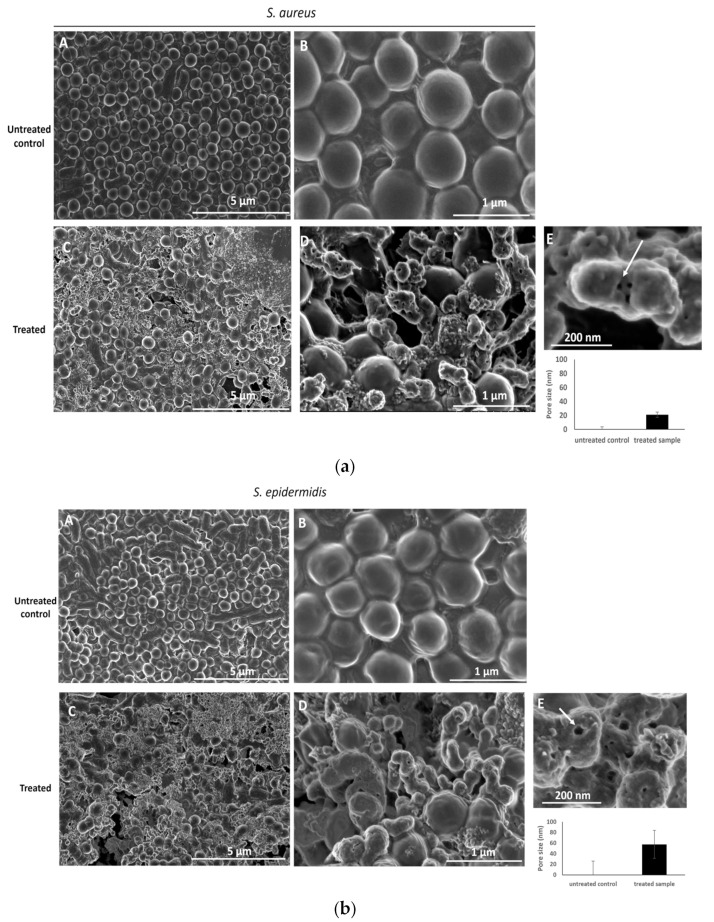
SEM images of methanolic extract from *Cynoglossum tubiflorus* leaf-treated (0.5 × MIC = 78 μg/mL) and untreated *S. aureus* (**a**) and *S. epidermidis* (**b**) bacteria. The SEM images were captured at 8 h post-treatment (**C**–**E**). Few treated cells appear with a normal shape and septum formation, while other cells are deformed, showing a rough surface, deformations, cell leakage, spillage of cell content, and debris after cell lysis, as well as pores of variable size ((**D**,**E**), white arrows indicate major structural changes and pores). SEM images of untreated *S. aureus* and *S. epidermidis* bacteria show normal morphology (**A**,**B**) with spewed-off contents after cell lysis.

HPLC-HESI-MS/MS analysis demonstrated that the methanolic extract is a promising source of secondary metabolites, including phenolic acids, flavonoids, lignans, and fatty acids. Notably, as shown in Table 5, this extract exhibited important antibacterial activity against Gram-positive bacteria compared to Gram-negative ones. This differential susceptibility forms the rationale for selecting the Gram-positive bacteria for the scanning electron microscopy assay in order to better understand the antibacterial activity of this extract. The scanning electron micrographs (SEM) of methanolic extract from *Cynoglossum tubiflorus* leaf-treated (at 0.5 × MIC = 78 μg/mL) *S. aureus* and *S*. *epidermidis* bacterial cells showed membrane distortions with surface depressions and change of the bacterial shape with dents compared to untreated cells (Figure 4) at time points 8 h post-incubation. The formation of pores on the cell surface inducing leakage was visible when the *S. aureus* cells were incubated for 8 h with a methanolic extract from *Cynoglossum tubiflorus* leaves at the same concentration (Figure 4(aE)). A similar ultrastructural change was observed at 78 μg/mL of methanolic extract from *Cynoglossum tubiflorus* leaf-treated cells after 8 h of incubation (Figure 4(bE)) as compared to untreated cells. The majority of the methanolic extract from *Cynoglossum tubiflorus* leaf-treated cells exhibited discernible holes on the cell surfaces (Figure 4(a,b—C,D)). Figure 4(aE,bE) clearly shows that exposure to methanolic extract from *Cynoglossum tubiflorus* leaves at 0.5 × MIC = 78 μg/mL caused damages to different extents to the *S. aureus* and *S. epidermidis* cell surfaces as the higher magnification of the SEM revealed the cell surface roughness, deep perforations or pores (white arrows, Figure 4(aE,bE)), along with complete lysis and shrinkage of several bacterial cells, often in clusters. The SEM observations also showed that pores induced by the treatment were varying in size between the treated *S. aureus* (average diameter 20 nm ± 3 nm) and *S. epidermidis* (average diameter 57 nm ± 26 nm), indicating a possible difference in the sensibility between these two Gram-positive bacteria to the treatment by methanolic extract from *Cynoglossum tubiflorus* leaves. 

Scanning electron microscopy was carried out to explore the major disruptions in the structure of Gram-positive *S. aureus* (ATCC 29213) and *S. epidermidis* (ATCC 14990) bacteria caused by the *Cynoglossum tubiflorus* leaf methanolic extract. The results revealed that bacterial cells exposed to the methanolic extract displayed morphological disorganization, including a rough cell wall, pore formation, and substantial cell content leakage, leading to bacterial cell lysis, in contrast to untreated bacteria, which maintained their normal structure. This observation may imply that methanolic extract could destroy the bacterial strains by damaging their cell walls. Such observations have been documented previously [48] in the case of plant extracts targeting the bacterial cell wall. Alternatively, some studies [49,50] described the destruction of pathogens through inhibition of protein and nucleic acid synthesis. Previous investigations have shown that many phytochemical compounds, namely tannins, triterpenes, flavonoids, alkaloids, sterols, and glucosinolates, can present potential antimicrobial activities [51]. Tannins, triterpenes, triterpenoids, and flavonoids from many plants can cause irreversible inhibition of several resistant bacterial strains, including *Staphylococcus* species, by altering the bacterial cell membrane permeability and fluidity, leading to a final damaging effect on the bacterial cell membranes [52,53,54,55,56,57,58]. The chemical analysis of the *Cynoglossum tubiflorus* leaf methanolic extract showed high contents of phenolic compounds, flavonoids, and lignans, such as chlorogenic acid, rosmarinic acid, cirsimaritin, kaempferol, and cinchonain Ib, described for their antibacterial activities [46,59,60,61,62,63]. For instance, kaempferol [64] has already been described as a compound that can destroy the bacterial membranes of *S. aureus* in a murine corneal infection model. Rosmarinic acid can also destroy *S. aureus* bacterial cells, with suggested impacts on the permeability of the cell membrane, protein metabolism, and DNA replication [65]. Furthermore, it has been demonstrated that chlorogenic acid can exert antibacterial activity against Gram-negative *Y. enterocolitica* by disrupting the cell membrane, increasing membrane permeability, and dysregulating many physiological pathways [66]. It has also shown antibacterial properties against Gram-positive bacteria like *Streptococcus pyogenes* [67]. The SEM observations clearly showed differential pore formation by *Cynoglossum tubiflorus* leaf extract between the treated *S. aureus* and *S. epidermidis*, with the latter presenting a three-fold bigger pore size. These two Gram-positive bacteria present similar, but not identical, structures of the cell wall. For instance, *S. epidermidis* presents a smaller repertoire of cell-wall-anchored surface proteins, known for their multiple functions in bacterial survival, than *S. aureus* [68,69], which may partially explain the difference in the response to the treatment with the methanolic extract from *Cynoglossum tubiflorus.*

### 2.7. Cytotoxic Effects

**Figure 5:**
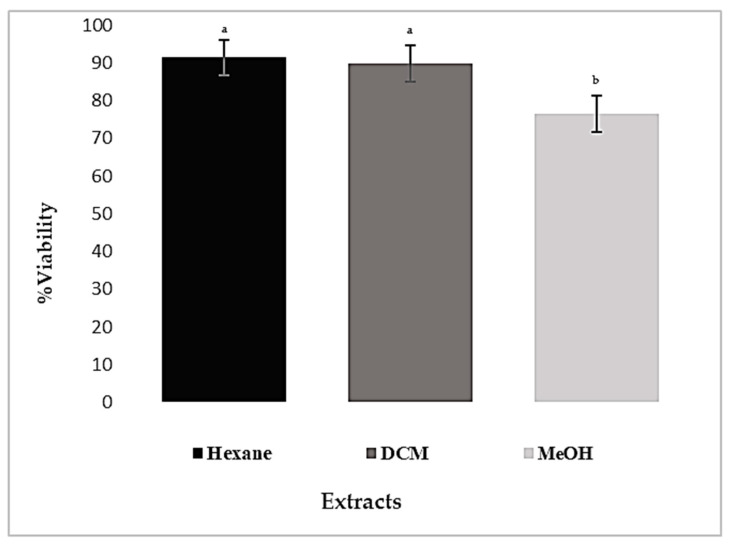
Viability of MRC-5 cells treated with the different extracts (600 µg/mL) for 72 h. Values are presented as mean ± SD (*n* = 3). The differences were analyzed using an ANOVA test for multiple comparisons using *p* < 0.05. ^a^: strong significance; ^b^: high model significance.

The cytotoxic effects of extracts on the human cell line MRC-5 were evaluated using the MTT assay after 72 h of continuous incubation. All the investigated extracts had no cytotoxic activity on MRC-5 cells at a concentration of 600 µg/mL. Moreover, extracts (hexane, DCM, and MeOH) did not reduce the cell survival of MRC-5 cells below 76%, as shown in Figure 5. This observation strongly suggests that these extracts did not exhibit any cytotoxic effect on the tested cell line. 

Phenolic compounds are abundant in plants and have garnered attention for their potential health benefits. They include simple phenols, flavonoids, lignans, tannins, xanthones, and coumarins. These compounds exhibit chemopreventive properties and have been studied for their cytotoxic effects. They can act as transcription factor inhibitors, which play a crucial role in protecting cells against oxidative stress and cancer progression, and clinical applications of polyphenols (phenolic compounds) as Nrf2 inhibitors are being investigated [70]. Clearly, given the in vitro antioxidant properties described here for the *Cynoglossum tubiflorus* extract, it seemed interesting to evaluate the cytotoxic properties of this phenolic-rich fraction on a human cell line (MRC-5). Indeed, the results of the MTT test showed no cytotoxic effect of the *Cynoglossum tubiflorus* fraction on the normal MRC-5 fibroblasts, used as a control cell line, after cell exposure for 72 h at all the concentrations assayed. The slightly different effects of individual extracts (hexane, DCM, and MeOH) on the survival of fibroblasts observed in this work are probably caused by the different compositions of biologically active compounds contained in the individual extracts. Previous studies have proved that plant extracts from leaves, also containing phenolic compounds, were able to reduce cell viability in tumor cell lines while preserving the viability of normal human cells or model organisms in lethality bioassays [71,72]. The observed outcomes may be attributed to an alternative mechanism of action, likely associated with the modulation of reactive oxygen species (ROS) levels and the disruption of the cellular redox state. Elevated ROS levels can trigger cell death processes, including apoptosis. Additionally, it is well established that many natural antioxidants function as effective free radical scavengers [73,74]. Given these properties, the presence of natural antioxidants in the methanolic extract of *Cynoglossum tubiflorus* could potentially mitigate the toxic effects of ROS. Our research findings indicate that the methanolic fraction may exert its effects by influencing the cellular redox balance, possibly by targeting intracellular ROS factors. The antioxidant properties are also linked to the enhanced activity of enzymes such as superoxide dismutase (SOD), catalase (CAT), and glutathione peroxidase, while simultaneously reducing the extent of lipid peroxidation. Additional scientific studies suggest that plant extracts may benefit fibroblasts by shielding them from oxidative harm. Other researchers have also affirmed that plant extracts positively impact normal cells by decreasing ROS, inflammatory cytokines, and matrix metalloproteinase-1 (MMP-1) expression [75]. Extracts from leaves have been shown to protect microglia cells, prevent cell death, and reduce the signs of neurological inflammation. Compared to other plant part extracts, leaf extracts exhibit stronger activity, likely due to a higher concentration of biologically active compounds in the leaves [76]. In the case of *Cynoglossum tubiflorus,* there is literature-based evidence for any cytotoxicity of the plant. Indeed, some studies, mostly based on the exploration of leaf extracts from other closely associated plants, such as *Cynoglossum lanceolatum Forsk*, *Cynoglossum cheirifolium*, *Cynoglossum creticum*, and *Cynoglossum zeylanicum*, have exclusively focused on the antioxidant properties of these plants, with no specific data on the cytotoxicity of leaf extracts of *Cynoglossum* spp. Other research studies of plants belonging to the same plant family, Boraginaceae, as *Cynoglossum tubiflorus* have shown that some plant members, such as *Onosma gmelinii,* have shown some cytotoxicity on Madin-Darby canine kidney (MDCK) cells, used as a model mammalian cell line. In that particular case, the CC_50_ values of root extracts of *Onosma gmelinii* obtained by different modes of extraction ranged from 7.5 to 236.6 μg/mL [77]. Another medicinal example from the family Boraginaceae, the comfrey (*Symphytum officinale* L.), is a plant usually employed for both external and internal use in different pathologies [78]. The toxicity potential of comfrey has been studied, but the detected toxicity is not directly linked to the phenolic compounds but rather to pyrrolizidine alkaloids such as symphytine and echimidine.

### 2.8. Selectivity Index Evaluation

The preliminary selectivity index (SI) evaluation of the three extracts (hexane, DCM, and MeOH) showed encouraging results. The SI was calculated to evaluate the toxicity of the compounds studied against normal cells and to predict their therapeutic potential, where a high selectivity index gives maximum antibacterial activity with minimal cell toxicity. All three extracts exhibit low cytotoxicity when tested at 600 µg/mL on healthy human cells of embryonic lung fibroblast origin, MRC-5, expressed as cytotoxic concentration (CC_50_) and percentage viability. The extracts also showed variable antibacterial activity, measured by their MIC (Table 5). Our results show that the MeOH extract in particular has an SI value (SI = CC_50_/MIC) higher than 1 for the Gram-positive screened bacteria (*S. aureus* and *S. epidermidis*) (Table 6).

### 2.9. Prediction by GUSAR (Based on PASS Prediction)

In the domain of drug design, using computational methods to predict compound toxicities enables rapid estimation of lethal doses in animals, thereby circumventing the need for actual animal experiments. In the context of pharmaceutical research and development, this specific stage plays a pivotal role within the intricate framework of drug discovery and subsequent transformation into an approved therapeutic product. The in silico prediction of rat acute toxicity for the investigated compounds (chlorogenic acid, rosmarinic acid, cirsimaritin, kaempferol, and cinchonain Ib) was conducted using the GUSAR (General Unrestricted Structure-Activity Relationships, version 16.0) software tool [79], which relies on the PASS (Prediction of Activity Spectra for Substances) technology. This quantitative in silico assessment encompassed four distinct routes of administration: oral, intravenous (IV), intraperitoneal (IP), and subcutaneous (SC). By analyzing the structural formula of the tested compounds, the predicted LD_50_ values for each route were compared, yielding insights into their acute rodent toxicity classification (Table 7). The calculation indicates that among the five tested compounds, there are no predicted super, extreme, or very high toxicities (class 1, class 2, or class 3) for all routes of administration. In light of the diverse routes of administration—namely intraperitoneal, oral, and subcutaneous—the five scrutinized compounds exhibited distinct toxicity profiles. Their estimated LD_50_ values, calculated through quantitative analysis, revealed nuanced patterns. Specifically, the compounds demonstrate moderate (classified as class 4), to slight toxicity (classified as class 5), and in some cases exhibit practically non-toxic behavior, suggesting minimal harm potential and a lower level of adverse effects.

### 2.10. Relationship between Measured Attributes

**Figure 6:**
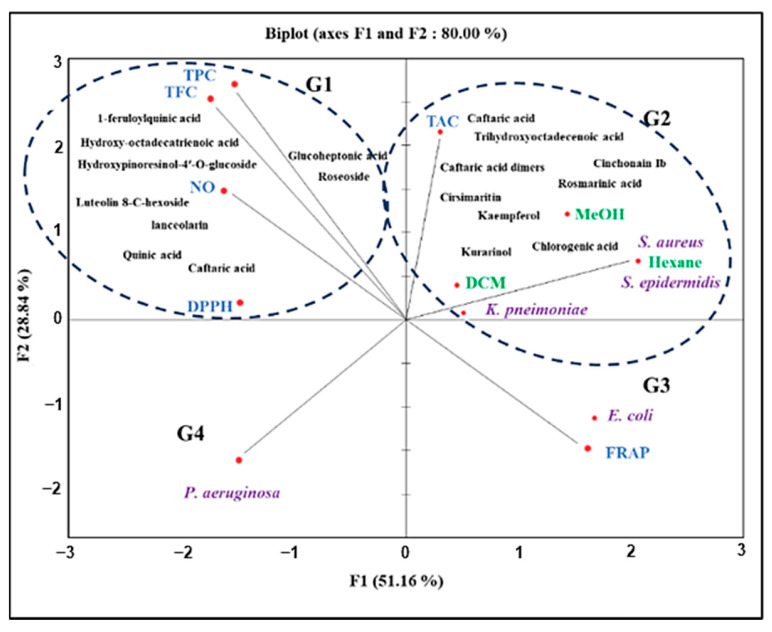
Correlation between the different extracts with the antioxidant effect (DPPH, NO, TAC, and FRAP), antibacterial activity, total phenolic contents (TPC), total flavonoid contents (TFC), and the most effective phenolics. Principal component analysis (PCA) biplot of components F1 and F2, explaining 80% of the variance in the sensory descriptive profiles of three extracts: DCM, hexane, and MeOH (and its compounds).

Our aim was to determine the relationship between phenolic compounds and antimicrobial and antioxidant activities (Figure 6). Several studies have shown a strong relationship between antioxidant activity and phenolics [80]. Our current study indicated a strong and positive correlation between TFC and TPC and NO (r = 0.963) and DPPH (r = 0.522) with some phenolic compounds like 1-feruloylquinic acid, hydroxy-octadecadienoic acid, roseoside, glucoheptonic acid, quinic acid, hydroxypinoresinol-4′-*O*-glucoside, luteolin 8-*C-*hexoside, and lanceolarin. It is recognized that flavonoids exert proton-donating capabilities and elevate radical scavenging activity, contingent upon specific structural characteristics, especially the position of hydroxyl groups [81]. Group G2 is mainly composed of hexane, DCM, and phenolic compounds in methanolic extracts such as chlolorogenic acid, rosmarinic acid, kurarinol, cirsimaritin, kaempferol, and cinchonain Ib, which correlate strictly positively with TAC (r = 0.567). In another case, in group G2, the best and most positive relationship was found between hexane and MeOH extracts and some bacteria, such as *Staphylococcus epidermidis* and *Staphylococcus aureus.* Moreover, principal components analysis (PCA) revealed that the three extracts (hexane, DCM, and MeOH) had the lowest correlation with *Escherichia coli* (G3) and no correlation with *Pseudomonas aeruginosa* (G4).

## 3. Materials and Methods

### 3.1. Chemicals and Reagents

All solvents, including dichloromethane, methanol, hexane, and ethanol, were purchased from Loba Chemie in Mumbai, India. Folin–Ciocalteu, Vitamin C, Quercetin, gallic acid, trichloroacetic acid, AlCl_3_, FeCl_3_, DPPH, Na_2_CO_3_, sodium phosphate, ammonium molybdate, and sulfuric acid were obtained from Sigma-Aldrich (commercial merck KGaA, darmstadt Germany). FBS, MTT, SEV, and MEM reagents were purchased from Sigma Aldrich in St. Quentin Fallavier, France. UV-visible analysis was recorded using equipment from Thermo Scientific in Saint-Herblain, France. Microscopy was performed with a Hitachi S-4800 microscope (Hitachi Europe, Issy les Moulineaux, France). HPLC-HESI-MS/MS analysis occurred using ThermoVanquish™ quaternary UHPLC system in-line with a Thermo Scientific™ Orbitrap ID-X™ Tribrid™ mass spectrometer (San José, Californie, US).

### 3.2. Plant Material

*Cynoglossum tubiflorus* was collected from Rouhia city, Siliana, Tunisia, in March 2020, with GPS coordinates 35.72531, 9.14916, and identified by Professor Mohamed Chaïeb [11], a botanist in the Department of Biology, Faculty of Sciences of Sfax, Tunisia. The leaves were collected from the lower regions of the plant species, and leaves at the top were avoided, where they may be exposed to harsher climatic conditions, as suggested by Situngu and Barker [82]. A voucher specimen numbered (LCSN156) was deposited at the Laboratory of Organic Chemistry, Natural Substances Team, Faculty of Sciences of Sfax, Tunisia. The leaves were dried at room temperature and stored in the dark until further use. 

### 3.3. Extraction

The dried leaves of *Cynoglossum tubiflorus* (500 g) were successively macerated using three organic solvents: hexane as the initial extraction solvent, followed by dichloromethane, and finally methanol. Extraction yields were obtained using Equation (1) as follows:

(1) $$Extraction\;Yield\left(\%\right)=\left(Dry\;extract\;weight/Dry\;starting\;material\;weight\right)\times 100$$

### 3.4. Phytochemical Composition

#### 3.4.1. Total Phenolic Content

The Folin–Ciocalteu method [83,84] was used to determine the total phenolic contents (TPC). Briefly, 1 mL of Folin–Ciocalteu reagent was added to a solution containing 1 mL of each extract (1 mg/mL) after 5 min. Next, 1 mL of saturated solution (Na_2_CO_3_/H_2_O) was added. The final mixture was incubated at 25 °C for 90 min, and then the absorbances were measured at 725 nm. Total phenolic content was expressed in milligrams of gallic acid (GAE) equivalent per gram of extract.

#### 3.4.2. Total Flavonoid Content

The method of Grati et al. [85]. was used to determine total flavonoid content. For this assay, quercetin was used as a standard. Moreover, 1 mL of aluminum trichloride (AlCl_3_) (2%) was added to 1 mL of sample (1 mg/mL), and the absorbance was recorded at 430 nm with a 15 min incubation at room temperature. Total flavonoid content was expressed in milligrams of quercetin equivalent (QE) per gram of extract.

### 3.5. Antioxidant Assays

#### 3.5.1. DPPH Assay

2,2-Diphenyl-1-picrylhydrazyl (DPPH) was used to assess the antiradical activity of the extracts. The scavenging activity of DPPH radicals was evaluated following the method of Fakhfakh et al. [86], with some modifications. Briefly, 2 mL of DPPH was mixed with different concentrations (0.063, 0.125, 0.250, 0.500, and 1.00 mg/mL) of plant extract and incubated in the dark for 30 min at room temperature. Optical densities (DO) were measured at 515 nm, using vitamin C as a positive control. The percentage of inhibition was calculated as follows:

(2) Inhibition (%) of DPPH radicals = [(A0−A1)/A0]×100

#### 3.5.2. Nitric Oxide Assay

The nitric oxide radical scavenging activity of *Cynoglossum tubiflorus* extracts was elaborated using the method described by ElAchaouia et al. [87]. Absorbances were measured at 550 nm. Vitamin C was used as a positive control. The percentage of NO in the extract was determined using the following equation:

(3) $$NO\left(\%\right)=\frac{A0-A1}{A0}\;*\;100$$

A0: Absorbance of control.
A1: Absorbance of extract.

#### 3.5.3. Ferric Reducing Antioxidant Power (FRAP) Assay

The ability of extracts to reduce Fe (III) to Fe (II) was assessed using the method of Barros et al. [88]. At different concentrations (0.063, 0.125, 0.250, 0.500, and 1.00 mg/mL), 1 mL of each exact was mixed with sodium phosphate (0.2 M) at pH 6 and potassium ferricyanide (1%). Mixtures were incubated at 50 °C for 20 min, then trichloroacetic acid (10%) was added, and the mixture was centrifuged for 10 min. After recuperation, the supernatant of each mixture was mingled with FeCl_3_ 0.1% in 2.5 mL of distilled water.

After 10 min of incubation, the absorbance was recorded at 593 nm.

#### 3.5.4. Total Antioxidant Capacity Assay

The total antioxidant capacity of extracts was assessed using the method of Rekik et al. [89]. For this test, 1 mL of reagent solution (sodium phosphate, ammonium molybdate, and sulfuric acid) was mixed with 0.1 mL of the sample and left at 95 °C for 1 h and 30 min. Absorbances were recorded at 695 nm using gallic acid as a standard.

### 3.6. Antibacterial Assay: Cells, Media, and Protocols

In this investigation, two Gram-positive bacteria, i.e., *Staphylococcus aureus* (ATCC 29213) and *Staphylococcus epidermidis* (ATCC 14990), and three Gram-negative bacteria, i.e., *Pseudomonas aeruginosa* (ATCC 27853), *Escherichia coli* (ATCC 25922), and *Klebsiella pneumoniae* (ATCC 700603), were used. All strains are routinely cultured at the PhotoNS-Bio platform of the L2CM laboratory (URM 7053 CNRS-UL, Vandoeuvre-lès-Nancy France). The minimal inhibitory concentration (MIC) was determined by the broth microdilution method according to the ISO 20771-1 guidelines [90]. Briefly, 50 µL of bacteria at 10^6^ CFU/mL in Mueller-Hinton broth, cation-adjusted (MHB-CA), are incubated with an equal volume of increasing concentrations of extract in MHB-CA. After 18 h of incubation at 35 °C, the MIC was determined as the lowest concentration of the extracts with no visible bacterial growth [91].

### 3.7. Electron Microscopy Assay

For the purposes of the electron microscopy assay, *Staphylococcus aureus* and *Staphylococcus epidermidis* bacteria, both in the exponential growth phase, were exposed to sub-minimal inhibitory concentrations (sub-MICs) of a methanolic extract derived from *Cynoglossum tubiflorus* leaves. Specifically, the concentration used was 0.5 times the minimum inhibitory concentration (MIC), which corresponds to 78 micrograms per milliliter (μg/mL). This treatment occurred over an 8-h period at a temperature of 35 °C. Untreated control groups were cultivated in standard Luria-Bertani (LB) growth medium. Subsequently, the bacterial specimens underwent fixation using a 30% ethanol solution. The samples were then subjected to progressive dehydration using a series of graded ethanol concentrations: 50%, 60%, 70%, 80%, 90%, and finally, two rounds of 100% (*v*/*v*) ethanol. Following dehydration, all specimens were carefully dried and coated with a thin film of palladium-gold using sputter deposition. All chemical reagents utilized were of electron microscopy grade and procured from the French branch of Delta Microscopies. The secondary electron images captured electron energies ranging from 1 keV to 2.5 keV.

### 3.8. Cytotoxicity Test: Cells, Media, and Protocols

The cytotoxicity of *Cynoglossum tubiflorus* leaf extracts was evaluated in the human cell line MRC-5 in culture for 72 h using an MTT assay, according to Khiralla et al. [92]. Cells were cultivated in Eagle’s minimum essential medium (MEM), complete with 10% heat-inactivated fetal bovine serum (FBS). The extracts were dissolved in DMSO, then diluted in MEM medium (at 2% FBS) at 1% final concentration, thus preventing a reduction in cell viability. Subsequently, 100 µL of medium with extract (600 µg/mL) were added to 10^4^ cells, reaching 80% confluence. The cells were grown at 37 °C in a humidified, 5% CO_2_ atmosphere. At 72 h post-treatment, the supernatants were discarded, and 100 µL of MTT (0.5 mg/mL) were added to each plate well. The plates were then incubated at 37 °C for 4 h. Absorbances were recorded at 570 nm using an ELISA plate reader.

### 3.9. Toxicity Properties Prediction by the GUSAR Software Tool (Based on PASS Prediction)

The compounds chlorogenic acid, rosmarinic acid, cirsimaritin, kaempferol, and cinchonain Ib were investigated for acute rat toxicity properties in silico. The GUSAR software tool was employed to forecast the acute toxicity (rodent models) of the antibacterial molecules present within the extract. Software was used to predict the acute toxicity in rodent models of the antibacterial compounds contained in the extract from *Cynoglossum tubiflorus* leaves [79]. This analysis relied on Quantitative Neighborhoods of Atoms (QNA) descriptors and the Prediction of Activity Spectra for Substances (PASS) algorithm. The results were correlated with the SYMYX MDL toxicity database. Subsequently, the tested compounds were classified based on their median lethal dose (LD_50_), which represents the dose lethal to 50% of experimental animals exposed to the compound. LD_50_ values were expressed as the weight of the chemical per unit of body weight (mg/kg). The classification followed the Gosselin, Smith, and Hodge scale, ranging from super toxic (class 1, LD50 ≤ 5 mg/kg), extremely toxic (class 2, 5 < LD_50_ ≤ 50 mg/kg), very toxic (class 3, 50 < LD_50_ ≤ 500 mg/kg), moderately toxic (class 4, 500 < LD_50_ ≤ 5.000 mg/kg), slightly toxic (class 5, 5.000 < LD_50_ ≤ 15.000 mg/kg), to practically non-toxic (LD_50_ > 15,000 mg/kg) [93]. 

### 3.10. HPLC-HESI-MS/MS

The methanolic extract of *Cynoglossum tubiflorus* leaves was investigated using a Thermo Scientific UHPLC-HRMS system. A C18 Alltima column (150 mm × 2.1 mm) maintained at 25 °C was delivered for the analysis. Solvents A and B, consisting of 0.1% formic acid in water (*v*/*v*) and 0.1% formic acid in methanol (*v*/*v*), respectively, were used. The elution gradient ranged from 5% to 95% of solvent B over 49 min, flowed by an isocratic step at 95% B for 5 min to wash the column, before returning to the initial composition of 5% B for 5 min to achieve equilibration. The mobile phase flowed at a rate of 0.2 mL/min with an injection volume of 20 µL. MS data acquisition was performed using Xcalibur v. 3.0 software (Thermo Scientific).

### 3.11. Statistical Analysis 

Data are obtained as means ± SD of triplicate (*n = 3*) assays. The significance of differences between assays was determined by ANOVA analysis at *p* < 0.05 using SPSS software version 20. The correlation between phenolic compounds in different extracts of *Cynoglossum tubiflorus* and antioxidant and antibacterial activities was determined by principal component analysis (PCA) using XL-Stat software version 2021.1.1.

## 4. Conclusions

The present paper deals for the first time with the identification of phenolic compounds in *Cynoglossum tubiflorus* leaf extracts. Thirty-two compounds belonging to phenolic acids, lignans, flavonoids, and fatty acids were identified. Furthermore, the investigation unveiled a correlation between the antioxidant activity of the methanolic extract and the elevated levels of phenolic and flavonoid compounds. These compounds demonstrated the most potent antioxidant effects in various assessments, including scavenging of DPPH free radicals, ferric reducing antioxidant power (FRAP), and total antioxidant capacity (TAC) tests. The results demonstrated the susceptibility of the bacteria to the different extracts, and the antimicrobial efficacy of the methanolic is likely to be linked to its richness of phenolic constituents, specifically chlorogenic acid, rosmarinic acid, cirsimaritin, kaempferol, cinchonain, etc. The scanning electron microscopy allowed the identification of significant structural changes in the Gram-positive bacteria resulting from exposure to the methanolic extract. These findings suggest that methanolic extract possesses the capacity to eradicate bacterial strains by disrupting their cell walls. *Cynoglossum tubiflorus* leaf extracts showed no cytotoxic effect on the human cell line MRC-5 at a high concentration (600 μg/mL). According to the obtained results, it can be concluded that *Cynoglossum tubiflorus* is a promising source of natural products with expressed antioxidant and antimicrobial activities.

## Figures and Tables

**Table 1:** Extraction yields of *Cynoglossum tubiflorus* leaves using different solvents.

Extracts	Yields (%)
Hexane	0.62 ± 0.50
DCM	1.04 ± 0.10
MeOH	1.68 ± 0.09

Values are presented as mean ± SD *(n* = 3).

**Table 2:** Total phenolic and flavonoid contents of *Cynoglossum tubiflorus* leaves.

Extracts	TPC (mg GAE/g DE)	TFC (mg QE/g DE)
Hexane	88.94 ± 0.171 ^c^	54.57 ± 0.586 ^c^
DCM	138.16 ± 0.114 ^b^	118.90 ± 0.352 ^b^
MeOH	254.34 ± 0.360 ^a^	211.59 ± 0.939 ^a^

TPC: total phenolic content; TFC: total flavonoid content; GAE: gallic acid equivalent; QE: quercetin equivalent; DE: dried extract. Values are presented as mean ± SD (*n* = 3). The differences were analyzed using an ANOVA test for multiple comparisons using *p* < 0.05. ^a^: strong significance; ^b^: high model significance; ^c^: low significance.

**Table 3:** Compounds from *Cynoglossum tubiflorus* leaves (MeOH extract) identified using HPLC-HESI-MS analyses (negative mode).

Compound	Tr (min)	Relative Abundance (%)	[M-H]^−^ (*m*/*z*)	HPLC-HESI-MS/MS (*m*/*z*)	Tentative Identification	Reference
**1**	1.55	0.07	225.0614	179/173/161	Glucoheptonic acid	[15]
**2**	1.64	0.07	341.1082	341(100)/191/179/161/119/101/89/71	Hexosyl hexose	[16]
**3**	1.66	1.53	191.0561	191(100)/173/127/109/93/85/59	Quinic acid	[17]
**4**	1.87	0.83	533.1730	191(100)	Quinic acid derivative	[18]
**5**	10.66	1.81	523.2076	523(100)/479/435/379/361/335/317/301/291/257/257/217/191/161/391	Caffeic acid dimers	[19]
**6**	10.74	1.42	499.1818	499/474/437/379/346/337/191/173/163(100)	3-*O*-Coumaroyl-5-caffeoylquinic acid	[20]
**7**	10.90	0.14	337.0922	163(100)/119/173/337	Coumaroylquinic acid isomers	[21]
**8**	11.72	0.02	515.1774	191(100)/353	1,5-Dicaffeoylquinic acid	[22]
**9**	11.92	1.04	503.2136	161(100)	Caffeoyl-fructosyl-glucose	[23]
**10**	12.37	1.32	353.0870	191(100)	Chlorogenic acid	[24]
**11**	14.09	1.67	431.1919	385(100)/223/153	Roseoside	[18]
**12**	15.98	1.25	431.1921	431/385(100)/226/179/173/153	Sinapic acid-*O*-hexoside	[25]
**13**	16.90	0.62	593.1519	473/383/353/325/297/503	6, 8-*C*-Dihexosylapigenin	[22]
**14**	20.34	2.6	367.1030	173/191(100)/367	1-Feruloylquinic acid	[22]
**15**	20.71	8.22	447.0946	357/327(100)/229/173	Luteolin-8-*C-*hexoside	[18]
**16**	20.74	0.02	535.1827	373(100)	Hydroxypinoresinol-4′-*O*-glucoside	[26]
**17**	21.55	0.02	451.1023	451/341(100)	Cinchonain Ib	[27]
**18**	22.89	1.79	359.0765	161(100)/179/197/135/123/72	Rosmarinic acid	[28]
**19**	23.84	0.48	463.0882	301(100)	Quercetin-3-*O*-glucoside	[29]
**20**	24.48	0.98	577.1569	577/283(100)/268	Lanceolarin	[30]
**21**	28.60	0.94	593.1313	593/284/285/255/227/151/145	Kaempferol-*O*-hexosyle-deoxyhexoside	[31]
**22**	29.24	0.07	285.0653	285/257/216/201/136/110/94	Kaempferol	[22]
**23**	30.63	0.04	313.0712	161(100)/133	Cirsimaritin	[32,33]
**24**	34.09	0.12	327.2169	327(100)/309/291/239/229/211/171	Oxo-dihydroxyoctadecenoic acid	[34,35]
**25**	35.98	6.52	329.2324	329(100)/229/211/171/139/127/99	Trihydroxyoctadecenoic acid	[36]
**26**	40.26	0.21	373.2592	373(100)/313/174	1-*O*-Galloyl-(2-*O*-acetyl)-glu	[37]
**27**	41.10	29.26	311.2222	311(100)/293/275/235/233/87	Caftaric acid	[38]
**28**	43.68	2.13	293.2120	293(100)/275/235	Hydroxy-octadecatrienoic	[36]
**29**	43.70	22.01	455.3015	455/161/311(100)/117/71	Kurarinol	[39]
**30**	45.33	8.53	295.2272	-	Hydroxy-octadecadienoic acid	[36]
**31**	49.78	2.13	277.2167	277	15,16-Dihydrotanshinone I	[36]
**32**	53.80	0.14	309.2793	309/289/199/103/98/60	Eicosaenoic acid	[36]

**Table 4:** Nitric oxide radical (NO) and DPPH scavenging activity of *Cynoglossum tubiflorus*.

Extracts	DPPH IC_50_ (mg/mL)	NO IC_50_ (mg/mL)
Hexane	0.383 ± 0.002 ^c^	0.268 ±0.009 ^c^
DCM	0.060 ± 0.001 ^b^	0.121 ±0.001 ^b^
MeOH	0.043 ± 0.001 ^a^	0.046 ±0.001 ^a^
Vitamin C	0.032 ± 0.001 ^a^	0.034 ±0.001 ^a^

Values are presented as mean ± SD (*n* = 3). The differences were analyzed using an ANOVA test for multiple comparisons using *p* < 0.05. ^a^: strong significance; ^b^: high model significance; ^c^: low significance.

**Table 5:** Summary of antibacterial properties of extracts (eight replicates per condition).

Microorganisms	Hexane MIC (µg/mL)	DCM MIC (µg/mL)	MeOH MIC (µg/mL)
*Staphylococcus aureus*	78	312	156
*Staphylococcus epidermidis*	78	312	156
*Pseudomonas aeruginosa*	625	-	-
*Escherichia coli*	312	625	625
*Klebsiella pneumoniae*	156	625	312

**Table 6:** Selectivity index of hexane, DCM, and MeOH extracts on five pathogenic bacteria. The cytotoxic concentration (CC_50_) used for the calculation of the SI is 600 µg/mL, allowing the survival of more than 50% of the host cells. For the compounds, values in bold are considered outstanding (SI > 1). *Sa* = *Staphylococcus aureus*; *Se* = *Staphylococcus epidermidis*; *Pa* = *Pseudomonas aeruginosa*; *Ec* = *Escherichia coli*; *Kp* = *Klebsiella pneumoniae*. A selectivity index value greater than 1 indicates that the compound is more toxic to pathogenic bacteria than to human cells. The greater the selectivity index value, the safer the compound is for the host, and values close to 10 are considered to be optimal.

Extracts	SI (CC_50_/MIC)				
	*Sa*	*Se*	*Pa*	*Ec*	*Kp*
Hexane	**7.69**	**7.69**	0.96	**1.92**	**3.85**
DCM	**1.92**	**1.92**	≤0.96	0.96	0.96
MeOH	**3.69**	**3.69**	≤0.96	0.96	**1.92**

**Table 7:** Prediction of acute rat toxicity by the GUSAR software tool.

	Compounds				
Acute Rat Toxicity Parameters	CA	RA	CM	K	CIb
Rat IP ^a^ LD_50_ (mg/kg)	1416.00	823.10	730.60	1163.00	527.00
Rat IV ^b^ LD_50_ (mg/kg)	229.30	323.60	1471.00	392.60	232.80
Rat Oral ^c^ LD_50_ (mg/kg)	3927.00	2994.00	2302.00	2183.00	804.70
Rat SC ^d^ LD_50_ (mg/kg)	343.20	826.30	3523.00	5938.00	426.00
Rat IP LD_50_ Classification	Non-toxic	Class 5	Class 5	Class 5	Class 5
Rat IV LD_50_ Classification	Class 4	Class 5	Non-toxic	Class 5	Class 4
Rat Oral LD_50_ Classification	Class 5	Class 5	Class 5	Class 5	Class 4
Rat SC LD_50_ Classification	Class 4	Class 4	Non-toxic	Non-toxic	Class 4

^a^ IP—intraperitoneal route of administration; ^b^ IV—intravenous route of administration; ^c^ Oral—oral route of administration; ^d^ SC—subcutaneous route of administration; CA—chlorogenic acid; RA—rosmarinic acid; CM—cirsimaritin, K—kaempferol; CIb—cinchonain Ib.

## Data Availability

Data are contained within the article.
